# Regenerative Rehabilitation for Stroke Recovery by Inducing Synergistic Effects of Cell Therapy and Neurorehabilitation on Motor Function: A Narrative Review of Pre-Clinical Studies

**DOI:** 10.3390/ijms21093135

**Published:** 2020-04-29

**Authors:** Akira Ito, Naoko Kubo, Nan Liang, Tomoki Aoyama, Hiroshi Kuroki

**Affiliations:** 1 Department of Motor Function Analysis, Human Health Sciences, Graduate School of Medicine, Kyoto University, Kyoto 606-8507, Japan; kubo.naoko.38e@st.kyoto-u.ac.jp (N.K.); kuroki.hiroshi.6s@kyoto-u.ac.jp (H.K.); 2 Cognitive Motor Neuroscience, Human Health Sciences, Graduate School of Medicine, Kyoto University, Kyoto 606-8507, Japan; liang.nan.3z@kyoto-u.ac.jp; 3 Department of Development and Rehabilitation of Motor Function, Human Health Sciences, Graduate School of Medicine, Kyoto University, Kyoto 606-8507, Japan; aoyama.tomoki.4e@kyoto-u.ac.jp

**Keywords:** rehabilitation, regenerative medicine, stroke, cell therapy, motor function, brain stimulation, epidural cortical stimulation, repetitive transcranial magnetic stimulation, transcranial direct current stimulation

## Abstract

Neurological diseases severely affect the quality of life of patients. Although existing treatments including rehabilitative therapy aim to facilitate the recovery of motor function, achieving complete recovery remains a challenge. In recent years, regenerative therapy has been considered as a potential candidate that could yield complete functional recovery. However, to achieve desirable results, integration of transplanted cells into neural networks and generation of appropriate microenvironments are essential. Furthermore, considering the nascent state of research in this area, we must understand certain aspects about regenerative therapy, including specific effects, nature of interaction when administered in combination with rehabilitative therapy (regenerative rehabilitation), and optimal conditions. Herein, we review the current status of research in the field of regenerative therapy, discuss the findings that could hold the key to resolving the challenges associated with regenerative rehabilitation, and outline the challenges to be addressed with future studies. The current state of research emphasizes the importance of determining the independent effect of regenerative and rehabilitative therapies before exploring their combined effects. Furthermore, the current review highlights the progression in the treatment perspective from a state of compensation of lost function to that of a possibility of complete functional recovery.

## 1. Introduction

Neurological diseases are major sources of burden to both patients and caregivers. Furthermore, the associated financial burden is immense, with major countries such as the U.S. spending over $800 billion each year on related expenses [1]. Among neurological diseases, stroke is not only the second leading cause of death worldwide, but also ranks highly among causes requiring long-term care. In the recent past, stroke often resulted in death; however, recent advances in medical care have helped medical practitioners save more patients than ever. Nonetheless, many patients still experience severe sequelae. While many treatment methods have been developed [2], there remains no approved and effective treatment method that facilitates the recovery of patients from subacute phase post-stroke, and rehabilitation is the only method that is known to contribute to the functional recovery and increase patient quality of life (QOL) [3].

In recent years, steady progress in the clinical applications of regenerative medicine has occurred for a variety of diseases. Cell transplantation has emerged as a promising treatment for stroke recovery. Integration of these cell into functional neural networks and generation of microenvironments in which they can work effectively are necessary to obtain desirable results. Furthermore, it is necessary to retrain the neural systems that were lost. Rehabilitative interventions are expected to play an important role in this process, which was been discussed in the Stem Cell Therapies as an Emerging Paradigm in Stroke (STEPS) guidelines [4,5]. However, many points remain that must be clarified before the application of regenerative medicine for the treatment of stroke becomes a reality: (i) the specific effects of rehabilitation in the context of cell therapy; (ii) potential synergistic or antagonistic effects of a combination of rehabilitation and cell therapy; and (iii) optimal form, frequency, and timing of its implementation, considering that rehabilitation is effective. The emerging field of regenerative rehabilitation is expected to answer such questions [6].

In the present review, we (i) discuss the current status of research on cell therapy for stroke recovery and challenges therein; (ii) outline the findings from the field of regenerative rehabilitation that could hold the key to unraveling these challenges; (iii) present the findings of past animal studies, evaluating the effects of approaches combining cell therapy and rehabilitation interventions; (iv) summarize the findings of pre-clinical research using brain stimulation, a method expected to yield drastic results when combined with cell therapy in the future; and (v) discuss future expectations and challenges faced in treating stroke with a combination of cell therapy and rehabilitation.

## 2. Cell Therapy for Stroke Recovery

**Figure 1:**
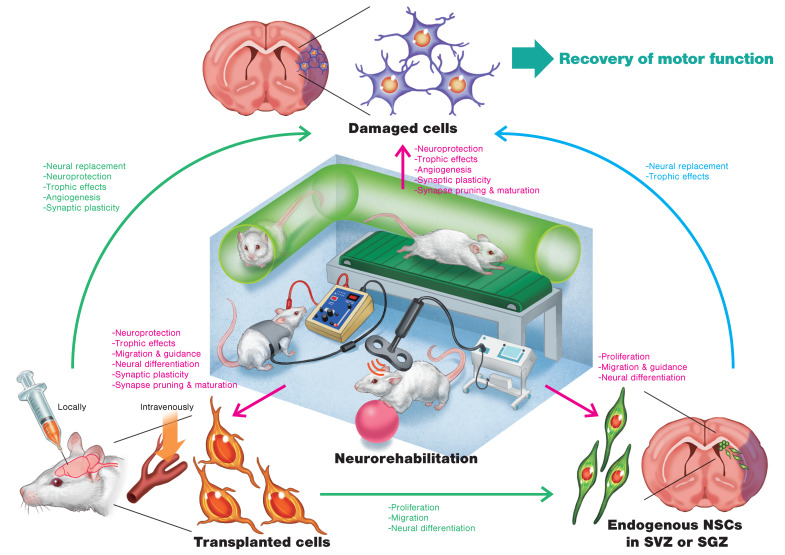
Schematic illustration of regenerative rehabilitation for stroke recovery in an animal model. Possible mechanisms of action related to cell therapy and neurorehabilitation are described. Transplanted cells affect endogenous neural stem cells (NSCs) in the subventricular zone (SVZ) or the subgranular zone (SGZ), as well as damaged cells in the infarct area. Neurorehabilitation such as treadmill exercise, enriched environment (EE) interventions, repetitive transcranial magnetic stimulation (rTMS), and transcranial direct current stimulation (tDCS) not only affects damaged cells but also affects endogenous NSCs and transplanted cells. Combining cell therapy and neurorehabilitation will be able to induce synergistic effects on motor function.

Cell transplantation therapy is a promising and appealing treatment method for stroke recovery [7]. Intraparenchymal and intravenous cell transplantation methods are the most frequently used methods in this area of research, while the cells used for transplantation include mesenchymal stem cells (MSCs), umbilical cord blood cells, pluripotent stem cells, and neural stem and progenitor cells (NSCs and NPCs) [7]. Neural stem cells in particular can integrate into neural networks to replace lost cells, and also regulate inflammation, secrete neurotrophic factors, and promote neurogenesis, thereby contributing to tissue repair (Figure 1) [8,9,10]. However, the many clinical trials, both completed and ongoing, concerning cell therapy for stroke recovery have yet to present definitive effects and treatment methods [11]. To establish cell therapy as a method for the treatment of stroke, we must overcome many remaining challenges. Specifically, we must establish which choices are optimal with regard to patient group, timing, location, frequency, cell type, and transplantation method. There are a particularly large number of persistent questions concerning survival of transplanted cells, engraftment and fate of cells, and maintenance of regular characteristics [12,13].

A study of the optimal time window for therapeutic intervention after brain injury by Peron et al. showed that transplantation of cells into the brain one week after injury is effective [14]. Meanwhile, Stroemer et al. reported that transplanting human NSCs (hNSCs) into post-stroke rats promoted significant behavioral recovery that is dependent upon the dose of cells [15]. In their comparative study of different cell transplantation sites, Kelly et al. demonstrated that transplanting cells at a site farther from the edge of the lesion increased cell survival [16]. In another study on transplantation sites, Smith et al. revealed that transplantation in the vicinity of the brain injury resulted in a better recovery than that in the cerebral ventricle [17]. Further, they demonstrated that a high engraftment degree of transplanted cells does not necessarily lead to functional recovery and that network reconstruction through localized engraftment and synapse formation is important. They also showed that most of the transplanted hNSCs differentiate into astrocytes, with only around 2% differentiating into neurons [17]. Angiogenesis and increased synaptic plasticity, as well as the promotion of communication between adjacent cells and the extracellular matrix due to astrocytes’ paracrine and juxtacrine effects, respectively, are thought to contribute to the functional recovery.

In addition to the transplantation site, the source of the transplanted cells is also important for the reconstruction of normal neural networks. When E14 embryonic motor cortical tissue was transplanted into the injury sites of mice with motor cortex injury, the tissue integrated successfully into the host motor cortex and was able to develop fibers projecting towards the sensorimotor cortex, the same as those seen in a healthy mouse [18]. Importantly, it has been demonstrated that synaptic inputs from the stroke-injured brain to transplanted neurons are functional [19], and neuronal activity in the transplanted cells might regulate motor behavior of the stroke-affected animals [20]. Interestingly, when E14 embryonic visual cortical tissue was transplanted into the injured motor cortex, axons grew predominantly toward the visual cortex, which is a stark contrast from the path of the motor cortical tissue [18,21]. These findings imply that not all embryonic cortical cells have the capacity to contribute to the reconstruction of a specific injured neural network and suggest that using pre-committed embryonic neurons helps read the molecular cues sent from the injury site and complete necessary actions. This means that cell-autonomous and environmental cues could regulate axonal extension after cell transplantation [22]. Therefore, brain organoids are expected to be plausible cell sources for transplantation. A study by Wang et al. exploring the effects of brain organoid transplantation in a rat model of middle cerebral artery occlusion (MCAO) found that cerebral infarct volume was greatly reduced and neuromotor function was improved [23]. The transplanted brain organoids imitated cortical development and supported motor-cortex-specific reconstruction. This study suggests the possibility that the transplanted brain organoids could differentiate across multiple systems, including the formation of neurotransmitter-related neurons and synapse connections with the host brain.

**Figure 2:**
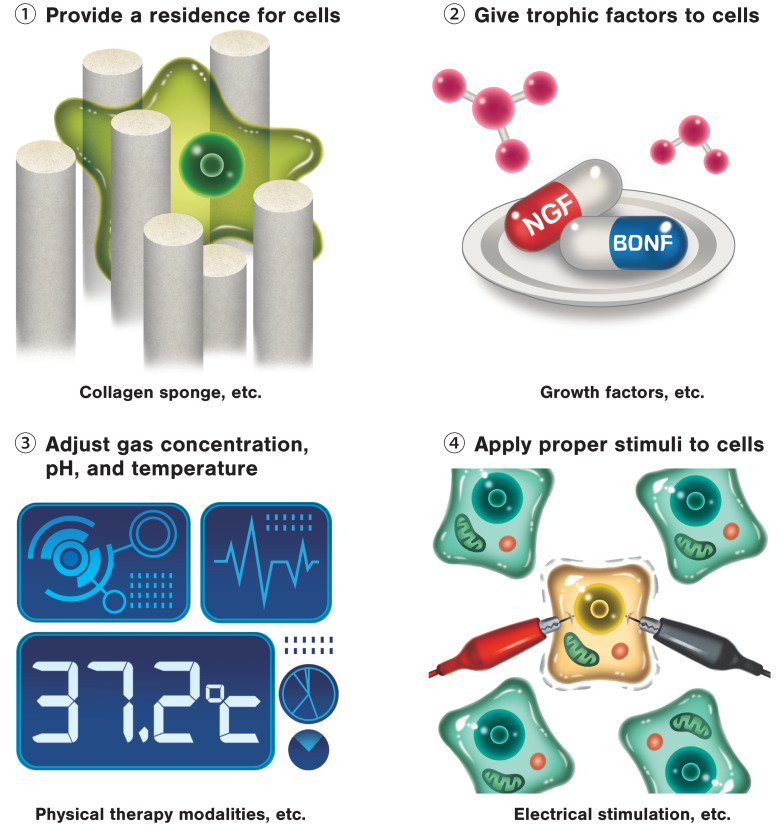
Adjustment of microenvironments is the key to success for cell therapy. Providing a residence for cells; providing sufficient nutrients; regulating gas concentration, pH, and temperature; and applying appropriate mechanical stress are considered essential for the success of cell therapy.

Studies on cell transplantation methods utilizing tissue engineering strategies are also making great strides. Because age is strongly involved in predicting the functional prognosis of patients with stroke, the ability to recreate microenvironments similar to those of young individuals holds the key to supporting the functional recovery [24]. Tissue engineering strategies are able to create regenerative environments. Micro devices and biomaterials are able to temporally control cells and small molecules such as growth factors, and can deliver these to the necessary site [25,26,27]. These are more easily able to mimic the appropriate microenvironments of transplanted cells, and therefore can improve survivability and promote differentiation of transplanted cells. Further, if a large section of cerebral parenchyma is lost, the substrates evoking the biological effects of therapy, such as rehabilitation, are also lost, meaning de novo functional tissue is necessary [28,29]. Therefore, optimizing the microenvironment of the transplantation site is considered essential for the success of cell therapy (Figure 2).

The above-mentioned findings demonstrate the continuous advances in current research and support the application of cell therapy as a promising treatment for stroke recovery. Establishing cells to be transplanted and the transplantation method, as well as further clarification of the mechanism of action of cell therapy, remain to be clarified for clinical application of cell therapy. Additionally, rehabilitation intervention post-stroke, discussed in further detail in subsequent sections, is one factor leading to uncontrollable outcome variation in clinical trials [4,5]. It is essential that further studies on the development of rehabilitation methods be carried out in order to establish cell therapy as a treatment for stroke recovery.

## 3. Regenerative Rehabilitation

Although expectations are high for cell therapy to be a completely new treatment method for many diseases, such methods unfortunately cannot be called universal at present. Cell therapy generally requires cell engraftment, survival, and differentiation into matured cells. Additionally, the transplanted cells need to reconstruct tissue form and must further be functionalized as a tissue or organ. However, there is limited research on post-transplantation therapy (i.e., rehabilitation research), and the lack of information on how to support engrafting, survival, differentiation, and functionalization after transplantation remains a challenge.

**Figure 3:**
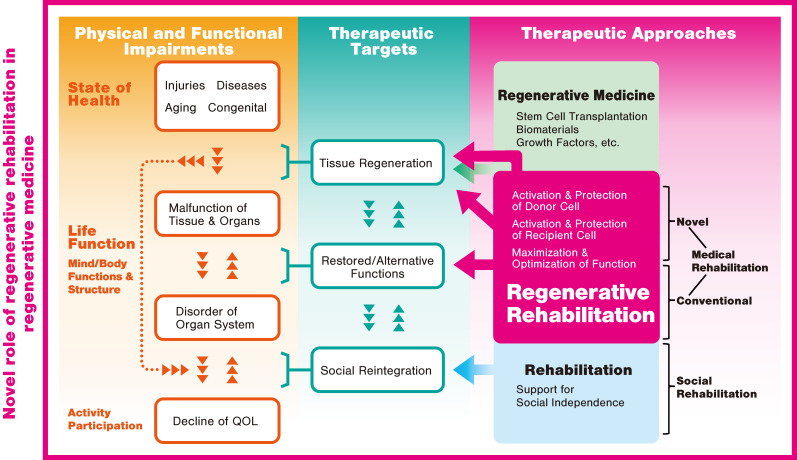
Schematic diagram of regenerative rehabilitation. Regenerative rehabilitation will play a novel role in regenerative medicine. QOL: quality of life.

The concept of regenerative rehabilitation was developed to solve this problem. Regenerative rehabilitation can be briefly defined as “the application of rehabilitation protocols and principles together with regenerative medicine therapeutics toward the goal of optimizing functional recovery through tissue regeneration, remodeling, or repair” [30]. The concept is characterized by the promotion of tissue regeneration and functional recovery through synergy with regenerative therapy techniques that are not generally incorporated into standard rehabilitation (Figure 3). It is believed that an effective combination of rehabilitation with regenerative medicine techniques can encourage the activation and preservation of transplanted (donor) cells, as well as prepare a niche and activate and preserve recipient (host) cells, thereby accelerating tissue regeneration. Regenerative rehabilitation further maximizes conventional rehabilitation approaches with consideration of the importance of enabling patients to reclaim their daily activities and return to society. Technical rehabilitation developments are also underway, owing the recent advances in robotics and similar fields [31,32,33].

### 3.1. Regenerative Rehabilitation for Stroke Recovery

Research on regenerative rehabilitation for stroke recovery is still in its infancy, and there is limited evidence of its effects. The therapeutic interaction of cell therapy and rehabilitation, not specifically for stroke, can be generally grouped into five categories: synergistic, additive, subadditive, antagonistic, and overlapping [34]. A synergistic interaction denotes an interaction in which the therapeutic effects of each method combine to yield a greater effect than the sum of individual effects. In an additive interaction, the effects of individual therapies do not influence one another, and the result is equivalent to the sum of effects of each separately. In subadditive interactions, the resultant effect is lower than the sum of the two therapies. In an antagonistic interaction, one therapy reduces the effectiveness of the other. Lastly, an overlapping interaction is an interaction in which the two therapeutic effects overlap, resulting in an effect equivalent to one of the therapies on its own. In the following subsection, we discuss the results of studies verifying the effects of combining cell therapy and rehabilitation intervention in animal models of stroke (Table 1).

### 3.2. Effects of Combining Intravenous Cell Transplantation and Rehabilitation

Several studies examined the effects of combining intravenous cell transplantation with rehabilitation intervention. Zhang et al. studied the therapeutic effects, particularly the neuroprotective effects, of a combination of MSC transplantation and treadmill exercise in MCAO model rats [39]. The results demonstrated that the number of apoptotic cells was significantly lower and the motor function was significantly higher in a synergistic manner in the combination group than in the single therapy groups. The combination group showed a significant increase in the number of engrafted MSCs and inhibition of cell death in both host and donor cells as a result of treadmill exercise. It is hypothesized that this anti-apoptotic effect involves the upregulation of 2 regulators of cell death, survivin, and bcl-2 [39]. In a similar study by Sasaki et al., combination therapy demonstrated a synergistic effect, significantly reducing infarct volume, increasing corpus callosum thickness, and inducing synaptogenesis, thereby increasing recovery of motor function [41]. A study by Zhao et al. revealed that combining mild therapeutic hypothermia (33 °C) and rat adipose-derived stem cells (rADSCs) improved functional recovery additively or synergistically through reduction of neuronal apoptosis and gliosis, promotion of angiogenesis, and reduction of the infiltration of innate immune cells in MCAO model rats [42]. Recently, Mu et al. investigated the effects of combining the intravenous transplantation of human adipose-tissue-derived MSCs (hADMSCs) and enriched environment (EE) interventions in MCAO model rats [43]. EE is a method of intervention in which animals are reared in a highly stimulating environment [44]. When enrichments such as novel and colorful objects or different types of activities are provided within an animal’s cage, the animal attempts to adapt to its environment, which has been found to lead to functional recovery in animal models of stroke [44]. Mu et al. reported that the combined therapy enhanced behavioral recovery, however the extent of angiogenesis or gliosis was not related to the behavioral recovery [43]. They mentioned that the results could be due to the loss of cells after xenogenic transplantation, the presence of spontaneous recovery and of a ceiling effect, and the complex study design, making it difficult to discriminate the add-on therapeutic effect.

Intravenous transplantation of MSCs or ADSCs has the potential to synergistically improve motor function when combined with rehabilitation intervention. Research suggests the involvement of various mechanisms such as cytoprotection, increase in synaptic plasticity, and angiogenesis in this improvement of motor function, although there remains some discrepancies. 

### 3.3. Effects of Combining Local Cell Transplantation and Rehabilitation

Multiple studies have reported the effects of combining local cell transplantation into the cerebral parenchyma with treadmill exercise or EE interventions.

A study by Seo et al. investigated the effects of combining treatment with transplantation of human adipose stem cells (hASCs) to the striatum and EE in hypoxic-ischemic brain injury model mice [38]. Their study demonstrated that the combination of therapies synergistically promoted functional recovery by strengthening the engrafting and neural differentiation of transplanted hASCs, inducing intrinsic neurogenesis and activating astrocytes through upregulation of fibroblast growth factor-2 (FGF-2). A more detailed study of the same group found that therapy combining hASC transplantation and EE promoted functional recovery as a result of synergistic upregulation of angiogenic factors, including FGF-2, vascular cell adhesion protein-1 (VCAM-1), matrix metalloproteinase-2 (MMP-2), angiopoietin-1 (ANGPT-1), and angiopoietin-2 (ANGPT-2) released from the activated astrocytes and concurrent intrinsic angiogenesis [40].

Hicks et al. published three papers examining the effects of combination therapy with EE using a variety of animal models of cerebral infarction and cells intended for transplantation [35,36,37]. In 2007, the group studied the effects of therapy combining EE and the transplantation of mouse subventricular zone (SVZ)-derived stem cells into the sensorimotor cortex and striatum in MCAO model rats [35]. Combination with EE significantly increased the distance of stem cell migration towards the infarct site and yielded recovery of motor function at an earlier stage. The study did not establish a treatment group receiving only EE; therefore, it is not clear whether cell transplantation and EE have a synergistic effect. The following year in 2008, Hicks et al. investigated the long-term (1 to 3 months) effects of the same combined therapy used in the previous study (mouse SVZ-derived stem cell transplantation and EE) [36]. Long-term observation revealed that EE has no effect on cell transplantation. The vast majority (~99%) of transplanted cells died within two months and cell survivability was found to be negatively correlated with microglial activation. Thus, it is possible that the transplanted cells were eliminated as a result of an immune rejection response. Hicks et al. conducted a third study to evaluate the effects of combining EE and transplantation of human embryonic stem cell (hESC)-derived NPCs using permanent distal MCAO model rats [37]. The method of immunosuppressant administration was changed from previous studies [35,36] to continuous administration via osmotic minipumps, but the transplanted cells were still eliminated by the host immune system. The cell transplantation only group showed a modest trend of motor function improvement, but no improvement was seen in the EE alone or combination therapy groups.

Verifying the effects of combining two therapies is only possible if the therapeutic effects of each of them are independently assured. We must continue to optimize the cell transplantation therapy method and the rehabilitation intervention method.

The above experiments produced different results, perhaps because of the fact that intraparenchymal cell transplantation is technically more difficult than intravenous transplantation. Other reasons could be the higher number of examined variables, as well as the use of different cells, which could be one of the cause of variability in outcomes. Before exploration of the effects of combination with rehabilitation can advance, it is essential to further standardize cell transplantation protocol.

Treatment with intraparenchymal cell transplantation therapy alone had limited effects on motor function recovery and required the addition of rehabilitation interventions [38,40]. On the contrary, cell transplantation was found to improve the regenerative environment through processes such as angiogenesis, and it is speculated that this synergistically increased the motor function recovery resulting from rehabilitation.

## 4. Brain Stimulation for Stroke Recovery

Brain stimulation has been studied as a method of neurorehabilitation for regulating and enhancing brain function [45]. Many studies have focused on epidural cortical stimulation (CS), in which electrodes are neurosurgically placed on the surface of the brain for stimulation; and noninvasive brain stimulation (NIBS) techniques, which included transcranial direct current stimulation (tDCS) and repetitive transcranial magnetic stimulation (rTMS). Recent years have seen enthusiastic research activity on stroke treatment using brain stimulation in both humans and laboratory animals with promising results [46,47,48]. Animal experiments are slowly revealing the effects of brain stimulation at the cellular level (Figure 1), and studies have reported the effects of combining these techniques with cell therapy. In the following sections, we discuss the findings of animal studies focusing on treating stroke with CS, rTMS, and tDCS.

### 4.1. Epidural Cortical Stimulation (CS)

Many animal experiments revealed that CS enhances the neural reorganization effects induced by rehabilitation training. In rat models of cerebral infarction, CS increased motor cortical evoked potentials in the stimulated region [49] and densities of dendrites and synapses in layer V [50,51]. CS research has not focused on rat models alone. One study on a squirrel monkey model found that CS promoted the re-emergence of movement representations [52]. We also know that the effects of CS are dependent on frequency, polarity, and other stimulus parameters [49,53]. Stimulating a wide section of the motor cortex is more effective than local stimulation [49,54]. The severity of the injury also influences the effects of CS. Specifically, it was found that a combination of CS and training exercises were not effective in improving functional recovery in a rat model of severe cerebral infarction [51]. The anatomical region that is injured is also thought to be associated with this result [55]. In other words, it has been hypothesized that corticospinal tract integrity is essential for CS to have an effect. The timing of initiation of CS is known to have an effect. Increased functional recovery resulting from CS could no longer be confirmed when therapeutic intervention was started as early as three months after the onset of cerebral infarction [56,57]. In addition, the effect of CS is time- and age-dependent [58]. Following these pre-clinical trial results, clinical trials using CS were carried out. Phase I and II clinical trials demonstrated the safety and effectiveness of the technique [59,60]. However, in the final stage of phase III clinical trial, CS did not reach the primary efficacy endpoint, and therefore it was found to be ineffective [61]. Subsequent studies identified the reason for this contradictory finding to be the use of suboptimal stimulus parameters relative to those discovered through animal experiments [61]. In addition to the CS stimulus parameters, issues with participant inclusion criteria were also noted. In animal experiments, most studies included subjects that exhibited an evoked response to stimulus from the electrodes. However, in the clinical trials, the proportion of subjects showing evoked response was 100% in phase I, 42% in phase II, and a mere 16% in phase III [61]. In other words, it is highly likely that these trials included subjects who were not responsive to CS. In fact, a subgroup analysis investigating the effects of CS in subjects who did show an evoked response found significant functional recovery in the CS group [62]. On the basis of these findings, we can conclude that we must approach clinical research with the aim of elucidating these findings through empirical research and seek to fully clarify targets of treatment based on this research.

### 4.2. Repetitive Transcranial Magnetic Stimulation (rTMS)

Repetitive transcranial magnetic stimulation (rTMS) is a stimulation method in which a sequence of magnetic stimulation is repeatedly administered to a region of the cortex. It has been demonstrated that this method is able to temporarily regulate the excitability of the stimulated cortex, and can thereby modulate neuroplasticity of the cerebral cortex [63]. Conventional rTMS modalities include high-frequency (>5 Hz) and low-frequency stimulation (⩽1 Hz) [64]. In the stimulated region, high-frequency rTMS generally increases motor cortex excitability and low-frequency rTMS reduces excitability. Many unknown aspects remain regarding the mechanism by which rTMS regulates the brain; however, the phenomena of long-term potentiation and long-term depression seem to be involved.

Recent in vitro and in vivo research has begun to clarify details of synaptic and cellular changes induced by rTMS [65,66,67,68]. As a non-pharmacological tool that can be used to support neurorehabilitation after stroke, rTMS is thought to have the potential to contribute to the clinical recovery of patients with stroke when used in combination with other traditional rehabilitation interventions [46].

Unlike clinical studies, most of the studies using animal models of stroke begin with the evaluation of the effects of rTMS in the acute phase. Below, we outline the effects of rTMS in animal models of stroke and the related mechanisms of action.

### 4.3. Effects of rTMS on Animal Models of Stroke

Most studies report the effects of rTMS in the acute phase, which begins within 24 hours after stroke. Ipsilesional high-frequency (20 Hz) rTMS that was started one hour after cerebral infarction reduced apoptosis and infarct volume and improved the neurological outcome [69]. Similarly, when ipsilesional high-frequency (10 Hz) rTMS was administered 24 hours after the onset of stroke, apoptosis in the hippocampus was reduced and neurogenesis increased [65]. Further, rTMS increased the proliferation of adult neural stem cells in the subventricular zone in both normal rats and MCAO model rats [70,71], while rTMS also led to the increased expression of c-Fos and brain-derived neurotrophic factor (BDNF) in the cortex, including the infarct area [66]. It has been speculated that rTMS may contribute to post-stroke functional recovery by elevating the expression of c-Fos—a marker of neuronal activity—resulting in the upregulation of BDNF. Similarly, high-frequency (10 Hz) rTMS that was administered in a mouse model of cerebral hemorrhage was found to reduce cerebral edema, improve motor function, and promote proliferation and neuronal differentiation of neural stem cells via the MAPK signaling pathway, as well as to suppress glial differentiation [67]. An in vitro study using ischemia and reperfusion model neurons also showed that repetitive magnetic stimulation (rMS) is effective [68]. While no beneficial effects were observed for low-frequency (0.5 Hz) rMS, high-frequency (10 Hz) rMS resulted in cell proliferation via activation of the extracellular signal-regulated kinases and AKT signaling pathways, as well as inhibition of apoptosis. Furthermore, high-frequency rMS enhanced the Ca2^+^-calmodulin-dependent protein kinase II (CaMKII)–cAMP-response element binding protein (CREB) signaling pathway, thereby promoting BDNF expression and synaptic plasticity [68].

The rTMS interventions resulted in beneficial effects, even when started 1 to 7 days after the onset of stroke. Administering high-frequency (10 Hz) rTMS 4 to 18 days after MCAO reduced apoptosis and improved motor function [72]. Ipsilesional high-frequency (20 Hz) rTMS initiated three days after MCAO promoted ipsilateral subventricular zone neurogenesis and increased the migration of neural precursor cells in the striatum near the infarction [73]. In vitro studies on the effects of rTMS on neural stem cell proliferation found the possible involvement of downregulation of p21, which accompanies the upregulation of miR-106b [74].

Interestingly, administering rTMS before transient ischemic attack has been found to induce ischemic tolerance, thus preventing neuronal cell death in the hippocampus [75]. This study suggests that rTMS has potential to be used as a pre-treatment in patients who are scheduled to undergo treatments that may cause transient ischemia.

These studies demonstrate the extremely wide range of effects of rTMS in animal models of stroke, including motor function recovery, reduction of infarct volume, mitigation of cerebral edema, anti-apoptosis, elevation of neurotrophic factors, facilitation of neurogenesis, promotion of proliferation and migration of neural precursor cells, and furtherance of neuronal differentiation and inhibition of glial differentiation.

### 4.4. Transcranial Direct Current Stimulation (tDCS)

Transcranial direct current stimulation (tDCS) is a noninvasive method used to stimulate the brain activity and modify the brain behavior by applying a weak direct current from outside the skull. Because the necessary equipment is highly safe and portable, tDCS has been actively studied in numerous conditions, including stroke, epilepsy, Alzheimer’s disease, and Parkinson’s disease [76]. While there is much to be understood about the mechanism of action, facilitating synaptic transmission through the release of noradrenaline and elevation of astrocyte calcium levels has been noted as one mode of action of tDCS [77]. Many polarity-dependent effects have also been reported. While cathodal stimulation temporarily inhibits cortical excitability, anodal stimulation temporarily increases cortical excitability [78]. Two primary methods of stimulation have been employed to improve its function in patients with stroke. In the first method, anodal stimulation was applied to an injured hemisphere exhibiting reduced cortical activity. In the second method, inhibitory cathodal stimulation was applied to a non-injured hemisphere, causing excess interhemispheric inhibition. However, these polarity-dependent effects were not fixed, and significant individual differences have been reported; therefore, caution is essential in interpreting these results [79,80].

Similar to studies on rTMS, several studies verified the effects of tDCS intervention using animal models of stroke at the beginning of the acute phase, unlike clinical studies. In the following section, we will outline the effects of tDCS in animal models of stroke and related mechanisms of action.

#### Effects of tDCS on Animal Models of Stroke

Cathodal tDCS administered immediately after MCAO reduced infarct volume [81]. Infarct volume was lower and motor function was better in MCAO model mice that underwent ipsilesional cathodal tDCS 4.5 hours after the onset than in those that underwent sham stimulation and anodal tDCS [82], as a result of decreased apoptosis, lessened edema, and reduced inflammation. Meanwhile, it has been shown that anodal tDCS may have adverse effects. For example, ipsilesional anodal tDCS has the potential to exacerbate inflammation and accelerate cerebral hemorrhage [82]. In another study, a combination of ipsilesional cathodal tDCS and peripheral sensory stimulation of the contralateral forelimb in stroke model mice significantly reduced infarct volume, improved peripheral circulation, inhibited microglia activation, and improved motor function [83]. In another study, tDCS that was started one day after MCAO significantly increased cortical dendritic spine density [84]. The above-mentioned studies offer evidence on the beneficial effects of cathodal tDCS applied to the ipsilesional hemisphere in the acute phase within 24 hours after stroke onset.

Studies have also found that tDCS initiated 1 to 7 days after the onset of stroke is useful. One study examining the effects of ipsilesional anodal tDCS initiated 1 or 7 days after infarction in MCAO model rats found motor function improvement in both starting conditions [85]. Expression of microtubule-associated protein-2 (MAP-2) and growth associated protein-43 (GAP-43) was correlated with this improvement. A study on the effects of ipsilesional cathodal tDCS and ipsilesional anodal tDCS initiated 3 days after infarction in MCAO model rats found that both polarities increased neurogenesis [86]. Further, cathodal tDCS was found to accelerate the genesis and migration of oligodendrocyte progenitor cells from the subventricular zone to the ischemic lesion [86].

Similarly to rTMS, these results confirm the wide range of effects of tDCS in animal models of stroke, including improvement of motor function, reduction of infarct volume, mitigation of cerebral edema, anti-apoptotic and anti-inflammatory effects, increase in dendritic spine density, neurogenesis, and facilitation of stem and progenitor cell migration.

As seen in the above examples, most studies found that the rTMS and tDCS groups showed a better post-stroke tissue preservation and functional improvement than did the control or sham stimulation groups. However, research methods used for animal experiments and clinical trials differ in several important ways. In most animal experiments, only stimulation of the lesioned hemisphere is studied, and most studies on rTMS used ipsilesional high-frequency stimulation. However, clinical studies have demonstrated the effectiveness of low-frequency stimulation of the contralesional hemisphere as well [46]. It will be essential for future animal experiments to further clarify the therapeutic effects of this treatment and its mechanisms of action. Additionally, as the intensive rehabilitation interventions used post-rTMS in clinical settings are largely unstudied in animal experiments, it will be necessary to examine the effects of combining NIBS and task-oriented rehabilitation interventions. Lastly, the rTMS and tDCS conditions used in animal experiments are often more drastic than those recommended for clinical studies [87,88]. Thus, there is a need for studies that include conditions within the range appropriate for clinical application.

### 4.5. Therapeutic Effects of Combining Cell Transplantation and Brain Stimulation

As noted above, brain stimulation not only reduces infarct volume, prevents apoptosis, increases neurotrophic factor levels, and improves synaptic plasticity, but also facilitates intrinsic neurogenesis and cell migration to the site of injury. Crossing these characteristics with those of cell transplantation therapy is expected to yield a synergistic effect (Figure 1). Although very few studies examined this aspect, in the following text we discuss relevant findings published to date (Table 2).

A study by Morimoto et al. investigated the effects of combining CS and transplantation of 2.5 × 10^5^ rat femur-derived MSCs to the left corpus callosum (contralesional) one day after infarction in right-MCAO model rats [89]. A cathode was placed in the ipsilesional frontal epidural space, and CS was implemented continuously at an intensity of 100 µA and a frequency of 100 Hz, 24 h per day, for two weeks immediately following transplantation. As a result, the combination group led to a significantly higher reduction in the infarct volume and recovery of the motor function than did the cell transplantation only group. The study did not use a CS therapy only group; therefore, the presence of synergistic effects is unclear. However, CS significantly increased the area and distance of migration towards the lesion of the MCSs transplanted into the contralesional corpus callosum. It was suggested that upregulation of stromal cell-derived factor-1α (SDF-1α) resulting from CS administration is associated with this ability to migrate. Therefore, electrical stimulation may be able to improve transplanted cell migration and guide cells to the target region to some extent [90]. Kremer et al. transplanted 6 × 10^5^ human dental pulp stem cells into the striatum and cortex of normal rats and performed low-frequency TMS on the hemisphere ipsilateral to the transplantation site every second day for two weeks, beginning two days after transplantation [91]. TMS was carried out at an intensity of 60% of the maximum output and a frequency of 0.2 Hz for 15 min under anesthesia. As a result, the number of transplanted cells dropped significantly and expression of the apoptosis marker Caspase-3 increased in the TMS group. The study did not perform neurological evaluations, making it difficult to determine whether cell transplantation itself was carried out effectively, but the study did suggest that the TMS stimulation condition, which is generally too strong in intensity, may have had an adverse effect on the transplanted cells. Lastly, Peng et al. studied the therapeutic effects of combining high-frequency rTMS with the transplantation of 2.5 × 10^5^ hNSCs into the striatum four days after infarction in MCAO model rats [92]. The rTMS was started the day after cell transplantation and was administered without anesthesia to the ipsilesional hemisphere at an intensity of 26% of the maximum output and a frequency of 10 Hz for a 3 s duration, 10 times, with an interval of 50 s between each administration (300 pulses/day). This combination of hNSCs and rTMS demonstrated a synergistic effect, increasing the motor function significantly more than either of the treatments alone. Further, a synergistic or additive effect because of the combination was also observed for neurogenesis in the ipsilesional SVZ. The underlying mechanism was suggested to involve the BDNF/TrkB signaling pathway because the upregulation of BDNF and elevation of phosphorylated TrkB/total TrkB were confirmed. Another interesting finding is that the combination of hNSC transplantation and rTMS is associated with a significantly lower proliferation rate of transplanted cells and higher rate of incidence of neuronal differentiation marker-positive cells than is hNSC transplantation alone. The rTMS may also have potential as a modality for facilitating neuronal differentiation in regenerative medicine.

The above-mentioned studies demonstrate that combining brain stimulation with cell transplantation can facilitate synergistic motor function recovery through the promotion of neuronal differentiation and migration of transplanted cells to the target area. As improper stimulation conditions can lead to negative effects, it will be essential to optimize stimulation parameters and consider factors such as injury site, transplanted cell type, and the timing and conditions of stimulation. Lastly, it may be possible to enhance the effects of cell therapy further by combining this technique with brain stimulation and intensive exercise therapy, which have already shown clinical results [46]. This will be a promising topic for future studies.

## 5. Conclusion and Future Perspectives

This paper discussed the effects of combining cell therapy and rehabilitation for stroke recovery. Many studies report findings that suggest synergistic effects and promising clinical applications of these therapeutic strategies, but it is necessary to accumulate further evidence in this regard. Furthermore, the methods of brain stimulation introduced here have an extremely wide range of effects for stroke treatment, and evidence suggesting the beneficial effects of their combination with cell therapy is gaining momentum. The impact of such evidence could drastically change the very concept of therapy for stroke recovery. In the recent past, it was thought that the central nervous system could not regenerate, and the focus of rehabilitation was on the ability to somehow compensate for the loss function using the function that remained. In addition to advances in neuroscience, new findings have been integrated into rehabilitation to give rise to the concept of neurorehabilitation. Methods such as constraint-induced movement therapy and task-oriented training, which attempt to facilitate changes in synaptic plasticity based on Hebb’s rule and activate reorganization of brain mapping, have been developed. With the recent advent of cell transplantation therapy, facilitating a marked suppression of inflammation, protection of cells and promotion of functional compensation have been achieved. The next goal is to achieve true regeneration of lost function based on these methods. To do so, discovering a method to induce the reconstruction of neural circuits from transplanted cells and endogenous neural stem cells will be the key. One particular challenge will be the distance required to be travelled by transplanted cells for the reconstruction of the corticospinal tract. In studies of natural stroke recovery, there is an association between better outcome and less corticospinal tract injury [93,94]. In addition, the extent of corticospinal tract injury predicts responses to neurorehabilitation, such as CS, rTMS, and robotic therapy after stroke [95,96,97]. Therefore, reconstruction of the corticospinal tract is important for the true regeneration of lost function. Regenerative medicine that incorporates appropriate rehabilitation may hold the secret to resolving this long-standing challenge.

**Figure 4:**
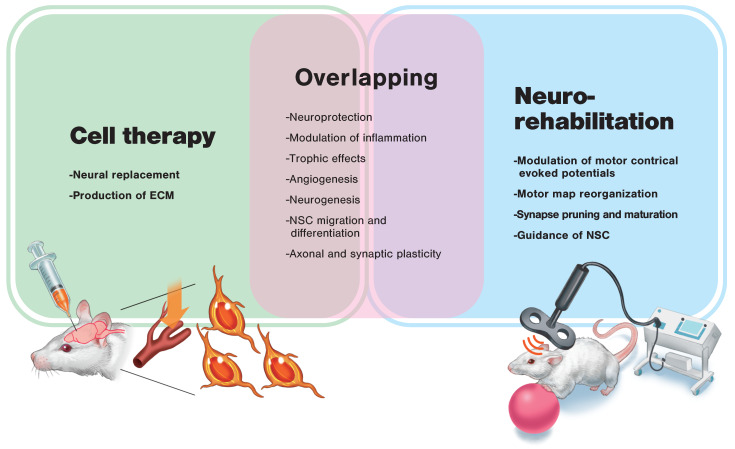
Possible mechanisms of action of cell therapy and neurorehabilitation for stroke recovery. Many possible mechanisms of action overlap between cell therapy and neurorehabilitation. In order to induce synergistic effects, each therapy’s specific mechanism of action needs to be considered. Appropriate timing and order of the application of these therapies is also a necessary consideration. ECM: extracellular matrix; NSC: neural stem cell.

Cell therapy and rehabilitation share much of the same extremely wide range of underlying mechanisms of action (Figure 4). However, cell therapy is considered essential to replenish the overwhelmingly deficient source of cells and create a regenerative environment. At the same time, the excessive number of neural connections, transiently brought about as a result of cell therapy, may cause malfunction [98]. At present, rehabilitation is the only way to achieve appropriate synaptic pruning according to Hebb’s rule. Thus, regenerative rehabilitation, which combines these two approaches, may be the key to regenerative therapy for stroke recovery. In addition, combined therapy, such as pharmacotherapy and rehabilitation, would be a promising strategy, meaning further research applying combination treatments are needed to foster stroke recovery [99]. 

The findings discussed in this paper demonstrate that interdisciplinary collaboration centering on regenerative medicine, rehabilitation medicine, and engineering is essential for regenerative rehabilitation research. Consequently, connecting these fields is a topic that must be addressed in the future [100].

Lastly, we should mention the translational issues of combining cell therapy and rehabilitation for stroke recovery. Pre-clinical research should take into account many potential factors that influence experimental stroke therapies, such as age, sex, and comorbidities [4,5]. Rehabilitative intervention is also thought to be a confounder. These make study designs complex and difficult to implement, resulting in low statistical power. Furthermore, budgetary constraints, the lack of adequate in vitro and in vivo models, and the enormous amount of time required to address the relevant factors impairs such attempts in research practice. In order to overcome these translational issues, the STEPS guideline has proposed establishing pre-clinical data-sharing platforms and multicenter pre-clinical trials (MCPTs) [5]. MCPTs are believed to enhance predictive value and statistical power in pre-clinical research and to provide a close-to-practice assessment of the potential treatment. Through applying these innovative attempts, we may be able to deliver the benefits of regenerative rehabilitation to patients.

## Figures and Tables

**Table 1:** Summary of therapeutic effects of combining cell therapy and rehabilitation (excluding brain stimulation) on motor function in animal models of stroke.

Reference	Model	Cell Therapy			Rehabilitation			Outcome	
		Transplanted Cell	Cell Mass and Location	Timing of Transplantation	Category	Onset	Duration	Interactive Effect on Motor Function	Mechanism
Hicks et al. [35] (2007)	MCAO in rats	Stem cells from mSVZ	8 × 10^5^ cells, ipsilateral sensorimotor cortex and striatum	7 days after MCAO	Enriched environment	8 days after MCAO	30 days	n.a.	↑Migration of transplanted cells
Hicks et al. [36] (2008)	Endothelin-1 induced MCAO in rats	Stem cells from mSVZ	8 × 10^5^ cells, ipsilateral sensorimotor cortex and striatum	7 days after MCAO	Enriched environment	8 days after MCAO	3 months	n.a.	Majority (~99%) of cells died within 2 months of transplantation
Hicks et al. [37] (2009)	dMCAO in rats	hESC-derived NPCs	8 × 10^5^ cells, ipsilateral sensorimotor cortex	7 days after dMCAO	Enriched environment	8 days after MCAO	66 days	No effect	Poor survival of transplanted cells
Seo et al. [38] (2013)	Hypoxic-ischemic brain injury in mice	hASCs	1 × 10^5^ cells, ipsilateral striatum	5 weeks after injury	Enriched environment	5 weeks after injury	8 weeks	Synergistic	↑Neurogenesis in striatum ↑Astrocytic activation
Zhang et al. [39] (2015)	MCAO in rats	rMSCs	3 × 10^6^ cells, intravenously	1 day after MCAO	Treadmill exercise (4 m/min for the 1st day, 8 m/min for the 2nd day, 12 m/min for the remaining days for 20 min, every day)	2 days after MCAO	12 days	Synergistic	↓Apoptosis
Cho et al. [40] (2016)	Hypoxic-ischemic brain injury in mice	hASCs	1 × 10^5^ cells, ipsilateral striatum	5 weeks after injury	Enriched environment	5 weeks after injury	8 weeks	Synergistic	↑Angiogenesis ↑Astrocytic activation
Sasaki et al. [41] (2016)	MCAO in rats	rMSCs	1 × 10^6^ cells, intravenously	6 hours after MCAO	Treadmill exercise (3 m/min for 20 min, every day for 1 week. Speed was increased by 3 m/min every week)	1 day after MCAO	34 days	Synergistic	↓Infarction volume ↑Corpus callosum thickness ↑Synaptogenesis
Zhao et al. [42] (2018)	MCAO in rats	rADSCs	2 × 10^6^ cells, intravenously	After common carotid artery reperfusion	Mild therapeutic hypothermia (33 °C)	During the ischemia	2 hours	Additive or synergistic	↓Infarction volume ↓Apoptosis ↑Angiogenesis ↓Glial scar formation ↓Inflammatory responses
Mu et al. [43] (2019)	MCAO in rats	hADMSCs	2 × 10^6^ cells, intravenously	2 or 7 days after MCAO	Enriched environment	2 days after MCAO	42 days	Overlapping or additive	→Infarction volume →Angiogenesis →Glial scar formation

MCAO: middle cerebral artery occlusion; hESC: human embryonic stem cell; NPCs: neural progenitor cells; mSVZ: mouse subventricular zone; dMCAO: distal middle cerebral artery occlusion; hASCs: human adipose stem cells; rMSCs: rat mesenchymal stem cells; rADSCs: rat adipose-derived stem cells; hADMSCs: human adipose-tissue-derived mesenchymal stem cells; n.a.: not applicable; ↑: up-regulation; ↓: down-regulation.

**Table 2:** Summary of therapeutic effects of combining cell therapy and brain stimulation on motor function in animal models.

Reference	Model	Cell Therapy			Rehabilitation			Outcome	
		Transplanted Cell	Cell Mass and Location	Timing of Transplantation	Category	Onset	Duration	Interactive Effect on Motor Function	Mechanism
Kremer et al. [91] (2016)	Normal rats	Human dental pulp stem cells	6 × 10^5^ cells, right cortex and striatum	n.a.	Ipsilateral TMS (60% of the maximal output, 0.2 Hz for 15 min, every 2nd day, beginning on day 3 post-transplantation)	2 days after transplantation	12 days	Antagonistic	↓Transplanted cell survival ↑Apoptosis
Morimoto et al. [89] (2018)	MCAO in rats	rMSCs	2.5 × 10^5^ cells, contralateral corpus callosum	1 day after MCAO	Ipsilesional cathodal CS (100 µA, 100 Hz, continuously)	1 day after MCAO	14 days	n.a.	↓Infarction volume ↑Transplanted cell migration ↑SDF-1α
Peng et al. [92] (2019)	MCAO in rats	hNSCs	2.5 × 10^5^ cells, ipsilateral striatum	4 days after MCAO	Ipsilesional rTMS (26% of the maximal output, 10 Hz, 300 pulses/day, every day)	5 days after MCAO	28 days	Synergistic	↑Neurogenesis in SVZ ↑BDNF/TrkB signaling pathway ↑Neural differentiation

TMS: transcranial magnetic stimulation; MCAO: middle cerebral artery occlusion; rMSCs: rat mesenchymal stem cells; CS: epidural cortical stimulation; SDF-1α: stromal cell-derived factor-1α; hNSCs: human neural stem cells; rTMS: repetitive transcranial magnetic stimulation; SVZ: subventricular zone; BDNF: brain-derived neurotrophic factor; TrkB: tropomyosin receptor kinase B; ↑: up-regulation; ↓: down-regulation.

## Abbreviations

QOL	Quality of life
STEPS	Stem cell therapies as an emerging paradigm in stroke
MSCs	Mesenchymal stem cells
NSCs	Neural stem cells
NPCs	Neural progenitor cells
MCAO	Middle cerebral artery occlusion
SVZ	Subventricular zone
SGZ	Subgranular zone
EE	Enriched environment
rTMS	Repetitive transcranial magnetic stimulation
tDCS	Transcranial direct current stimulation
rADSCs	Rat adipose-derived stem cells
hADMSCs	Human adipose-tissue-derived mesenchymal stem cells
hASCs	Human adipose stem cells
FGF-2	Fibroblast growth factor-2
VCAM-1	Vascular cell adhesion protein-1
MMP-2	Matrix metalloproteinase-2
ANGPT-1	Angiopoietin-1
ANGPT-2	Angiopoietin-2
hESC	Human embryonic stem cell
CS	Epidural cortical stimulation
NIBS	Noninvasive brain stimulation
BDNF	Brain-derived neurotrophic factor
MAPK	Mitogen-activated Protein Kinase
cAMP	Cyclic adenosine monophosphate
MAP-2	Microtubule-associated protein-2
GAP-43	Growth associated protein-43
SDF-1α	Stromal cell-derived factor-1α
MCPTs	Multicenter pre-clinical trials
